# Life Threatening Complication during Treatment of Erysipelas due to Undiagnosed Ischemia of the Calf

**DOI:** 10.1155/2009/306969

**Published:** 2009-12-21

**Authors:** Tomasz Brzeziński, Tomasz Ostrowski, Maciej Skórski

**Affiliations:** Department of General and Thoracic Surgery, Medical University of Warsaw, St. Banacha 1a, 02-097 Warsaw, Poland

## Abstract

Erysipelas is a superficial skin infection due to streptococci strains, which usually responds well to conservative treatment. Coexisting undiagnosed ischemia of the extremity may lead to severe complications. 57-year-old man developed large, circumflex ulceration of his right calf within two weeks before the admission after three-month treatment of erysipelas. Computer angiography showed chronic occlusion of the superficial femoral artery and the above knee popliteal artery. Rapid debridement of the wound took control over the infection. Patient required complex vascular procedure which allowed to prepare the ulcer for meshed skin grafts. Patient was discharged home on 64th hospital day with completely healed ulcer.

## 1. Introduction

Erysipelas is a superficial skin infection caused by Streptococci diagnosed in dermatologic and surgical outpatient clinics. It is successfully treated with antibiotics and some relief of symptoms can be observed even after 48 hours [1]. Mortality rate does not exceed 0.5% and is due to organ failure [1]. Small ulcers can appear when the illness is localized on lower extremities but surgical intervention is seldom necessary. Ischemia of the calf predisposes to gangrene and ulcer formation which can *develop to giant proportions*. Septic shock may lead to patient death if surgical treatment is not undertaken in time.

## 2. Case History

**Figure 1:**
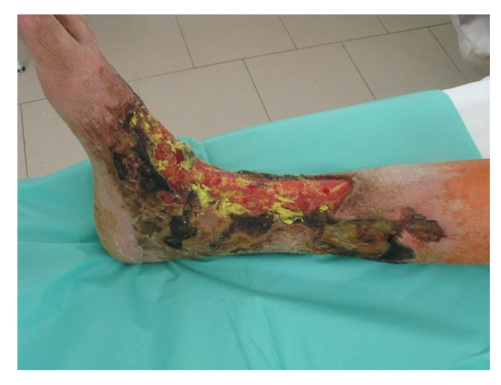
The view of the calf one week after the debridement: granulation is clearly visible with shrinking of the ulceration and remaining necrosis of the skin.

**Figure 2:**
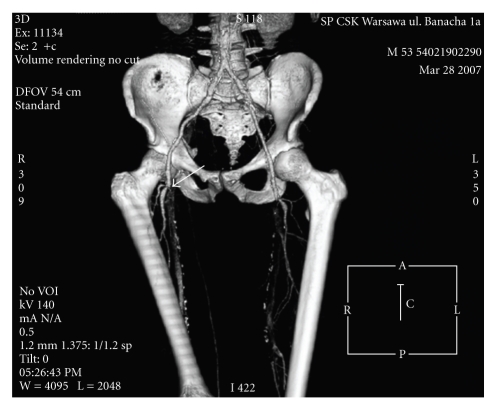
Computer angiography presenting arteries of the right lower extremity. An arrow shows the bifurcation of femoral artery and occlusion of superficial femoral one. Deep femoral artery is well developed. Common iliac artery and external iliac one seem to be normal.

**Figure 3:**
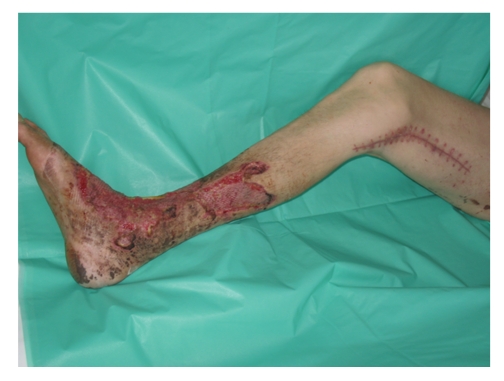
The view of the calf at the end of the treatment. Skin grafts nearly completely healed. Postoperative wound also healed, visible above the knee.

A 57-year-old male patient was admitted to our ward on a duty because of large circumflex ulcer of his right calf. On admission this patient was tachycardic with a temperature of 39 degrees centigrade, chills, and severe pain requiring narcotic pain killer. The circumflex ulceration started from the upper mid of the foot and reached upper one third of the calf. The remaining skin was necrotic and healthy margins were undermined with pus. The underlaying fascia disclosed muscles in patches. The smell was characteristic for severe inflammation. The patient also had a history of intermittent claudication and smoking of 30 cigarettes daily for more than 30 years. On examination there was no pulse in the popliteal fossa. *First report of the patient's current complaints was made 3 months earlier in dermatology clinic when he presented with painful, red swelling on his calf*. On that time erysipelas was diagnosed and he received antibiotics. He has been consulted several times by a dermatologist because of adverse course of his illness, small ulcer and pain. Sudden worsening appeared about two weeks before admission. In our ward the bacteria culture was taken and revealed strains of Beta Streptococcus and Enterobacter cloacae. Patient was first treated with Ciprofloxacin and then according to the antibiogram. Dedridement of the wound was performed under general anesthesia immediately and wet sponges with Octenisept, Octenidinum dihydrochloricum + Phenoxyethanolum (0,10 g + 2,00 g). Schulke GmbH, Germany, and 10% saline were continued. The preliminary result was good: body temperature returned to normal, granulation appeared in the wound, and the exudate was minimal (see Figure 1). Computer angiography revealed occlusion of superficial femoral artery and the above knee popliteal artery. Two arteries were patent on the calf (see Figure 2). Patient underwent elective vascular operation comprising of endarterectomy of the above knee popliteal artery with venous patch-plasty and femoro-popliteal bypass 6 mm PTFE, Polytetrafuroethylene, with above knee distal anastomosis performed just above the venous patch. Postoperative wounds healed very well. Vascular conduit improved granulation so much that it covered the whole ulceration; only remaining necrosis required excision. 28 days later the whole wound was covered with intermediate meshed split-thickness skin grafts expanded in a 1.5 : 1 ratio. The complete ingrowth was observed within 2 weeks (see Figure 3). Postoperative ankle-brachial index, the comparison of mean arterial pressure between foot arteries and brachial artery in horizontal position, was normal and close to 1.0. On the 64th hospital day the patient was discharged home with completely healed ulcer.

## 3. Discussion

Erysipelas is a superficial skin infection due to Steptococci which nowadays is localized on legs in most cases. Apart from Streptococci group A, a nongroup A Streptococci and other strains are main isolated pathogens although Enterobacter cloacae in our patient seems to be rather a superinfection. Staphylococcus aureus may play the role in bullous erysipelas with MRSA strains present [2]. Some medical conditions like diabetes, venous insufficiency, HIV infection, nephrotic syndrome, and alcohol abuse may predispose to erysipelas but it is doubtful whether they predispose to complications.

Erysipelas itself gives wide undermining of subcutaneous tissue which is very characteristic but even in severe cases like bullous erysipelas big ulcers are rare. Superficial necrosis and leg ulcer complicate 3%–12% of cases [1]. Ischemic calf creates completely different situation especially when the condition is not recognized early. As we know it predisposes to each kind of infection so that patients with severe and critical limb ischemia may present with ulcers of different sizes and localizations. The rapidly developing necrosis and ulceration within two weeks before admission could be due to necrotizing fascitis when gangrene tends to follow in multiple sites. Infections with widespread thrombosis and vascular necrosis of involved skin are two major factors in pathogenesis of gangrene [3]. Early debridement can prevent the spread of necrosis what proved to be very effective in our case. The vascular situation of this patient was good because two patent arteries on the calf provided good run-off. Local conditions on the ulcerated calf did not allow to perform the surgical incision below the knee because of high possibility of wound infection. So this changed the strategy of operation and endarterectomy of popliteal artery with patch plasty had to be done from supragenicular access to create conditions for the above-knee distal anastomosis of femoro-popliteal bypass. It is known that both venous and artificial femoropopliteal bypasses present with much higher patency rate when distal anastomosis is done above the knee joint [4].

The use of artificial vascular conduits is usually avoided at the presence of active inflammation on the operated limb [4]. In our case politetrafuoroethylene bypass seemed justified because of the lack of proper big saphenous vein on both legs [4, 5]. Ankle-brachial index is a useful and simple method for the assessment of limb ischemia. Normal values range between 0.8 and 1.0. Values of 0.5 and lower are usually due to occlusion of superficial femoral artery. Healing of ulcers on ischemic extremity without vascular procedure, classic operation, or endovascular treatment is very difficult or even impossible and may lead to amputation. *In literature the procedures concerning the ulcer itself, debridement, and skin grafts placing seem to increase the effectiveness of the whole treatment and allow to achieve a leg ulcer healing in combination with vascular operation* [6, 7].
